# Prevalence of cardio-metabolic multi-morbidity and associated risk factors in a population-based sample of South Africans

**DOI:** 10.1093/eurpub/ckac129.293

**Published:** 2022-10-25

**Authors:** N-B Kandala, R Sewpaul, AD Mbewu, AF Fagbamigbe, SP Reddy

**Affiliations:** 1 Division of Epidemiology and Biostatistics, University of the Witwatersrand, Johannesburg, South Africa; 2 Division of Health Sciences, Warwick University, Coventry, UK

## Abstract

**Objectives:**

Cardio-metabolic multi-morbidity (CM), the co-existence of two or more cardio-metabolic disorders in the same person, is rapidly increasing. We examined the prevalence and risk factors associated with CM in a population-based sample of South African adults.

**Study design:**

Data were analyzed on individuals aged ≥15 years from the South African National Health and Nutrition Examination Survey (SANHANES), a cross sectional population-based survey conducted in 2011-2012.

**Methods:**

CM was defined as having ≥2 of hypertension, diabetes, stroke and angina. Multivariable logistic regression was used to investigate the sociodemographic and modifiable risk factors associated with CM.

**Results:**

Of the 3832 individuals analyzed, the mean age was 40.8 years (S.D. 18.3), 64.5% were female and 18% were ≥60 years. The prevalence of CM was 10.5%. The most prevalent CM cluster was hypertension and diabetes (7.3%), followed by hypertension and angina (2.6%) and hypertension and stroke (1.9%). Of the individuals with diabetes, nearly three quarters had multi-morbidity from co-occurring hypertension, angina and/or stroke and of those with hypertension, 30% had co-occurring diabetes, angina and/or stroke. Age (30-44 years Adjusted Odds Ratio (AOR) = 2.68, 95% CI: 1.15-6.26), 45-59 years AOR = 16.32 (7.38-36.06), 60-74 years AOR = 40.14 (17.86-90.19), and ≥75 years AOR = 49.54 (19.25-127.50) compared with 15-29 years); Indian ethnicity (AOR = 2.58 (1.1-6.04) compared with black African ethnicity), overweight (AOR = 2.73 (1.84-4.07)) and obesity (AOR = 4.20 (2.75-6.40)) compared with normal or underweight) were associated with increased odds of CM.

**Conclusions:**

A tenth of South Africans have two or more cardio-metabolic conditions. The findings call for immediate prioritization of prevention, screening and management of cardio-metabolic conditions and their risk factors to avert large scale health care costs and adverse health outcomes associated with multi-morbidity.

